# The Microbiological Quality of Raw Ovine Milk in the Banat Region of Romania with a Focus on *Escherichia coli* and Its Pathogenic Potential and Antimicrobial Resistance

**DOI:** 10.3390/vetsci11110562

**Published:** 2024-11-13

**Authors:** Răzvan-Dragoș Roșu, Adriana Morar, Alexandra Ban-Cucerzan, Mirela Imre, Khalid Ibrahim Sallam, Al-Ashmawy A. Maha, Samir Mohammed Abd-Elghany, Sebastian Alexandru Popa, Răzvan-Tudor Pătrînjan, Doru Morar, Kálmán Imre

**Affiliations:** 1 Faculty of Veterinary Medicine, University of Life Sciences “King Mihai I” from Timişoara, 300645 Timișoara, Romania; razvan.rosu@usvt.ro (R.-D.R.); alexandracucerzan@usvt.ro (A.B.-C.); sebastian.popa@usvt.ro (S.A.P.); razvan.patrinjan.fmv@usvt.ro (R.-T.P.); dorumorar@usvt.ro (D.M.); kalmanimre@usvt.ro (K.I.); 2 Faculty of Veterinary Medicine, Mansoura University, 35516 Mansoura, Egypt; khalidsallam@mans.edu.eg (K.I.S.); mahaalashmawy@mans.edu.eg (A.-A.A.M.); drsamir@mans.edu.eg (S.M.A.-E.)

**Keywords:** microbiology, sheep milk, *Escherichia coli*, antimicrobial resistance

## Abstract

This study investigated the bacteriological quality of raw ovine milk produced by farms located in the Banat region of Romania. Additionally, the pathogenic potential and antimicrobial resistance of the isolated *Escherichia coli* strains were evaluated. A total of 95.8% (69/72) of the screened bulk tank milk samples, collected at the farm level immediately after milking, demonstrated appropriate total aerobic mesophilic bacteria (TMB) counts, varying from 3.32 to 6.09 log10 CFU/mL. However, 4.2% (3/72) of the samples were above the regulatory limit of 6.18 log10 CFU/mL. *E. coli* was identified in 66.6% of the examined samples, and from the total number (*n* = 48) of isolates, 18.8% harbored the *stx*2 gene, highlighting pathogenic potential. Antimicrobial susceptibility testing with the Vitek2 system of the isolated *E. coli* strains revealed resistance against ampicillin (45.8%), gentamicin (20.8%), ticarcillin–clavulanic acid (18.8%), cephalexin (18.8%), amoxicillin–clavulanic acid (8.3%), and trimethoprim–sulfamethoxazole (2.1%). Additionally, 64.6% of the strains showed intermediate resistance against amoxicillin–clavulanic acid, while no resistance was recorded against imipenem. Five (18.5%) strains were multidrug-resistant. This study’s results underline hygienic sanitary deficiencies during the milking phase and indicate that raw ovine milk can be contaminated with pathogenic and multidrug-resistant *E. coli* strains, highlighting a potential risk to public health. Further studies are encouraged to better understand the risk posed to the consumer via the consumption of ovine milk and derived products.

## Simple Summary

This investigation describes the results of the analysis of the microbiological quality of raw ovine milk in the Banat region of Romania, emphasizing monitoring the human infective potential and antimicrobial resistance of the isolated *Escherichia coli* strains. Overall, 4.2% (3/72) of the processed bulk tank milk samples exceeded the regulatory limit (6.18 log10 CFU/mL) for total aerobic mesophilic bacteria imposed by European Union legislation. *E. coli* was detected in 66.6% of the processed samples. Of the 48 isolated strains, 18.8% demonstrated pathogenic potential, and 18.5% were multidrug-resistant. Resistance was found against ampicillin (45.8%), gentamicin (20.8%), ticarcillin–clavulanic acid (18.8%), cephalexin (18.8%), amoxicillin–clavulanic acid (8.3%), and trimethoprim–sulfamethoxazole (2.1%), but all strains were susceptible to imipenem. This study provides important hygienic quality and safety data on raw ovine milk in Romania.

## 1. Introduction

Dairy sheep breeding is a traditional activity in Romania, with sheep being the second dairy animal species after cows. The total annual milk production was estimated at approximately 15,678 tons in 2022 in Romania (according to the latest available data provided by the Romanian Central Statistical Office) [1], making it the third most important ovine milk-producing country within the European Union. It is well known that most ovine milk, in unpasteurized form, is used to obtain different cheese assortments, achieved in small-scale integrated family farms or at the industrial scale [2]. The quality of the obtained products is closely related to the microbiological load of the raw ovine milk [3]. In this regard, the milk from healthy ovine mammary glands contains a limited number of germs (up to 6.0 log_10_ CFU/mL) [4], consisting especially of beneficial lactic acid bacteria that have probiotic potential and are responsible for dairy fermentations [5]. However, contamination can occur with unwanted spoilage (e.g., *Pseudomonas*, *Enterococcus*, or *Proteus*) or pathogenic microorganisms (e.g., pathogenic *Escherichia coli* strains, *Staphylococcus aureus*, or *Listeria monocytogenes*) during milking, handling, and storage from different sources (e.g., the udders of infected animals, milking equipment, the hands of the milkers, the udder surface, the air, or the environment), with a serious impact on the hygienic quality and biological safety of the derived products [6].

*Escherichia coli*, the main coliform species of the genus *Escherichia* from the Enterobacteriaceae family, is a Gram-negative, rod-shaped, and facultative anaerobic bacteria [7]. This microorganism is recognized as a commensal bacteria of the gastrointestinal tract of warm-blooded animals, from where it is excreted together with fecal matter, contaminating the external environment [8]. Hence, in the case of milk-producing animals, failures in the implementation of adequate hygiene practices during milking can result in the contamination of the raw milk and the subsequently derived products [2,9]. Most *E. coli* strains, as commensal bacteria, are considered non-pathogenic. However, the implication of some pathotypes (e.g., enterohemorrhagic, enterotoxigenic, enteroinvasive, enteropathogenic, enteroaggregative, and diffusely adherent strains) in several healthcare-associated diarrheal foodborne outbreaks has been frequently described in the scientific literature [10]. EHEC strains can produce *Shiga* toxins, with pronounced pathogenic effects on human health, consisting of bloody diarrhea, thrombotic thrombocytopenic purpura, and hemolytic uremic syndrome [11]. The occurrence of extraintestinal infections (e.g., urinary tract, neonatal meningitis, or sepsis) associated with *E. coli* pathotypes is also well documented [7].

Over the last few decades, the year-by-year increase in antimicrobial resistance (AMR) has become one of the most worrying universal threats in both the animal and public health sectors. In the case of food-producing animals, this phenomenon is linked to the overuse and misuse of veterinary drugs for either prophylactic or curative purposes. Additionally, the appearance and spread of the AMR phenomenon lead to the selection of multidrug-resistant bacteria at the gut level among pathogenic and commensal strains [9,12,13].

In a representative study, Tawfick et al. [8] demonstrated the propensity of *E. coli* (in either commensal or pathogen form) to serve as a reservoir for transmissible genetic elements (e.g., integrons, transposons, bacteriophages, or plasmids) accountable for genetic data transfer between bacterial strains at the gut microbiota level or capable of chromosomal mutations gaining resistance traits. Due to these properties, *E. coli* is considered an indicator or sentinel of the overall AMR profile of food-producing animal-origin bacterial strains with public health importance in a localized population [14]. In addition, *E. coli* is considered a frequently implicated bacteria in the transmission of AMR genes to humans via food matrices [15].

Taking this into consideration, this investigation aimed to provide information on the microbiological quality and appearance of *E. coli* strains in raw ovine milk obtained in the Banat region, Southwestern Romania, focusing on the evidence of virulence markers associated with enterohemorrhagic strains and their AMR profile determination.

## 2. Materials and Methods

### 2.1. Study Location and Sampling

This investigation was conducted between April and May of 2023 and 2024 during the main lactation season of the ewes in Timiș County, the Banat region, Romania. The screened area comprises the largest ovine population at the county level, with 732,981 heads (7.04% of the country’s ovine population) reared on approximately 2438 exploitations (data provided by the Sanitary Veterinary Directorate of Timiș County; reference date: April 2024). The required minimum sample size was computed according to the formula provided by Cochran [16], considering an expected prevalence of 75% [2], with a 95% confidence interval. Accordingly, 72 randomly selected dairy ewe farms whose owners agreed to participate in this study were enrolled. The investigated flocks were constituted especially from autochthonous breeds (e.g., Țurcană, Țigaie, and Merino), kept outdoors in a grazing system during the daytime, and had a size ranging between 120 and 2500 individuals. Each farm was investigated once during the study period, and the sampling was carried out after the early morning milking. The ewes were milked manually, without pre-milking sanitation or disinfection of teats, in traditionally arranged milking spaces within sheepfolds. Manual milking is regarded as an ancient task that is still largely performed at the countrywide level, and only a limited number of ewe dairies have adopted modern automated dairy equipment. According to the recommendation of the national legislation, the milk samples (~500 mL) for laboratory analyses were aseptically collected in sterile glass containers at the end of milking directly from the milk storage equipment used by the farms. The samples were delivered immediately under refrigerated conditions (≤4 °C) to the Microbiological Risk Assessment Laboratory of the Faculty of Veterinary Medicine, Timișoara, wherein the microbiological analysis was performed within the next two hours upon their arrival.

### 2.2. Bacteriological Examinations

Firstly, the microbiological examination of the milk specimens targeted the determination of the total aerobic mesophilic bacteria (TMB) counts, which develop at 30 °C, following the described steps in the international standard EN ISO 4833-1:2013 [17]. Briefly, serial dilutions in peptone buffer, starting from 1 milliliter (mL) of raw milk samples, were prepared up to a dilution of 10^−4^. Next, 1 mL from each dilution was transferred in duplicate into separate sterile plastic Petri dishes. Then, up to 15 mL of melted and cooled (<45 °C) nonselective nutrient agar medium was poured. The resulting mixture was incubated at 30 °C for a period of 72 h. Subsequently, the plates that presented between 15 and 300 grown colonies were counted [17], and the resulting colony-forming unit (CFU) numbers were transformed into log values.

The *E. coli* was detected and isolated according to the steps described by the International Organization and Standardization (ISO) no. 16649-2/2007 protocol, with slight amendments [18]. Briefly, 10-fold serial dilutions (10^−1^ to 10^−3^) using sterile peptone water (0.5%) were achieved. Next, 1 mL from each sample dilution was transferred in duplicate into individual disposable sterile plastic Petri dishes (Thermo Fischer Scientific, Bucharest, Romania). Subsequently, approximately 15 mL of the liquefied and tempered (~45 °C) tryptone bile with X-glucuronide (TBX) agar (Oxoid, Basingstoke, Hampshire, UK), which included 5-bromo-4-cloro-3-indolil-β-D-glucoronate as a chromogenic selective medium, was poured into each dish containing the diluted milk sample, mixed by rotating the dish, and then left to solidify. Then, the mixture was inverted and incubated aerobically in two subsequent steps (firstly, at 37 °C for 4 h, followed by 43.5 °C for 24 h). The grown samples from the 10^−3^ dilution, which produced typical blue–green-colored likely *E. coli* colonies, were subjected to Gram staining, and their number was accounted for accordingly [18]. The Vitek2 system (bioMérieux, Marcy l’Etoile, France) was utilized to identify the isolates at the species level, based on the testing of their complete biochemical properties and using the Vitek 2^®^ ID-GN (bioMérieux, Marcy l’Etoile, France) specific card. The reference strain *E. coli* ATCC 25922 was included as a control.

### 2.3. Molecular Characterization of E. coli Isolates

One *E. coli* isolate from each of the positive samples (*n* = 48) was subjected to molecular characterization. The DNA extraction was performed using a commercially available PureLink^TM^ Genomic DNA Mini Kit (Invitrogen™, Carlsbad, CA, USA) as per the manufacturer’s recommendations. In the first stage, all of the selected *E. coli* isolates were molecularly confirmed based on the evidence of the *tuf* (212bp) gene characteristic for *E. coli* and using the specific Tecol553 (5′-TGGGAGCGAGAAAATCCTG-3′) and TEcol754 (5′-CAGTACAGGTAGTAGACTTTTCTG-3′) primer set and cycling conditions as previously indicated by Maheux et al. [19]. Subsequently, screening for the presence of two typical *Shiga* toxin-producing genes characteristic of enterohemorrhagic *E. coli* strains, namely, *stx*1 (302bp) and *stx*2 (516bp), was performed. The specific primers used for the PCR reactions were F: 5′-CGC TGA ATG TCA TTC GCT CTG C-3′ and R: 5′-CGT GGT ATA GCT ACT GTC ACC-3′ for the amplification of the *stx*1 gene and F: 5′-CTT CGG TAT CCT ATT CCC GG-3′ and R: 5′-CTG CTG TGA CAG TGA CAA AAC GC-3′ for the amplification of the *stx*2 gene, according to the cycling conditions designed by Blanco et al. [20]. Within each PCR reaction, the *E. coli* O157 ATCC35401 reference strain was used as a positive control. The negative control consisted of nuclease-free ultrapure water. The resultant PCR products along with a DNA molecular weight marker (Bioline^®^, UK Ltd., London, UK) were separated by running on a 1.8% agarose gel marked with Midori Green (Nippon Genetics^®^; Europe, Gmbh, Düren, Germany). The molecular weight of the amplicons was visualized, estimated, and captured under a gel UV photo documentation system (UVP^®^, LLC, Upland, CA, USA).

### 2.4. Antimicrobial Susceptibility Testing of E. coli Strains

Vitek2 equipment (bioMérieux, Marcy l’Etoile, France), which automatically quantified the AMR of the tested *E. coli* strains (*n* = 48), was used. The AST-GN96 livestock-specific card was used, testing a total of seven antimicrobials belonging to five classes, namely, β-lactams (ampicillin [AMP; tested concentrations: 4, 8, 32], amoxicillin–clavulanic acid [AMC; 4/2, 16/8, 32/16], and ticarcillin–clavulanate [TIM; 8/2, 32/2, 64/2]); aminoglycosides (gentamicin [GEN; 4, 16, 32]); carbapenems (imipenem [IMP; 1, 2, 6, 12]); cephalosporins (cefalexin [LEX; 8, 32, 64]); and sulfonamides (trimethoprim–sulfamethoxazole [SXT; 1/19, 4/76, 16/304]). The selection strategy of the tested antimicrobials was based on the following considerations: AMP, AMC, GEN, LEX, and SXT were tested as regularly used drugs in food-producing animals in Romania (unpublished questionnaire results), while TIM and IMP were tested as antimicrobials whose use in food-producing animals is strongly advised against and considered as last-resort drugs for treating bacterial infections in humans. The isolates were classified as resistant, susceptible, or demonstrating intermediate resistance to the tested antimicrobials. The European Committee on Antimicrobial Susceptibility Testing (EUCAST) guidelines were used to establish the resistance breakpoints of the isolates [21]. The *E. coli* ATCC 25922 strain was used as an internal quality control.

### 2.5. Statistical Analysis

Statistical analysis was carried out using the SPSS ver. 23 software program (IBM, SPSS Advanced Statistics, IBM Corp., Armonk, NY, USA). Differences in the mean values of the TMB and *E. coli* counts according to the sampling months of the calendar years were interpreted using a one-way ANOVA test. Significance was assigned at *p* < 0.05.

## 3. Results

Table 1 summarizes the results of the microbiological and molecular investigations during the study period. The results of the bacteriological quality monitoring of raw sheep milk demonstrated that all analyzed samples harbored a certain level of aerobic mesophilic bacteria, with minimum, maximum, and average values of 3.32, 7.22, and 4.99 log_10_ CFU/mL, respectively. The TMB level exceeded the limit of 6.18 log_10_ CFU/mL imposed by the legislation [22,23] on 3 (4.17%; 95% CI 1.43–11.5) out of the total 72 farms involved in this study, with recorded values of 6.53, 6.87, and 7.22 log_10_ CFU/mL, respectively.

The occurrence of *E. coli* in the raw milk samples, depending on the investigation period (years and months), and the recorded values (minimum, maximum, and mean values) are presented in Table 1. Overall, 48 (66.6%; 95% CI 55.2–76.5) of the raw milk samples were contaminated with *E. coli*. Most of the samples (*n* = 32) harbored between 2.0 and 3.0 log_10_ CFU/mL of *E. coli*, while another 10 and 6 samples expressed contamination levels lower than 2.0 log_10_ CFU/mL and higher than 3.0 log_10_ CFU/mL, respectively.

No statistically significant differences were observed between the distribution of the mean values of TMB and *E. coli* counts according to the sampling months of 2023. However, in 2024, the mean value of the TMB count was significantly higher (*p* < 0.05) in May than in April, but the recorded values for the means of the *E. coli* counts between these months did not significantly differ (*p* > 0.05).

Molecular characterization of the biochemically confirmed *E. coli* strains (*n* = 48) was successfully achieved in all cases. The screening of two typical Shiga toxin-producing genes specific to enterohemorrhagic *E. coli* strains showed positive results in nine (18.8%; 95% CI: 10.2–31.9) cases for the *stx*2 gene, but no isolates harbored the *stx*1 gene.

The AMR patterns of the tested *E. coli* isolates (*n* = 48) to seven antimicrobial agents revealed resistance, in descending order, to AMP (45.8%), GEN (20.8%), TIM (18.8%), LEX (18.8%), AMC (8.3%), and SXT (2.1%). Notably, 64.6% of the tested strains expressed intermediate resistance levels against AMC. No resistance was recorded against IMP. Out of the 27 strains that expressed resistance to at least one drug, 5 (18.5%) were multidrug-resistant (Table 2).

## 4. Discussion

The TMB count is considered the most reliable indicator for determining the bacteriological quality of raw ovine milk, reflecting the sanitary and hygienic conditions of milking (e.g., animal hygiene conditions and the hygiene of milkers, the milking parlor, or equipment) and storage (e.g., containers or utensils). The mesophilic aerobes grow between 20 and 45 °C, and their amount is particularly significant when milk processing does not include thermal treatment [22]. In agreement with regulation (EC) no. 853/2004, the TMB count of raw ovine milk that will undergo heat treatment, determined at 30 °C, must not exceed 6.18 log_10_ CFU/mL (1.5 × 10^6^ CFU/mL), computed as the geometric mean over two months, with at least two sample analyses in each month. Nevertheless, the recommended limit for TMB, in the case of ovine milk processed without pasteurization, can be a maximum of 5.70 log_10_ CFU/mL (5.0 × 10^5^ CFU/mL) [23].

In this study, the recorded TMB values for raw ovine milk produced by 95.8% (69/72) of the investigated farms did not exceed the limit imposed by EU legislation, ranging from 3.32 to 6.09 log_10_ CFU/mL. However, the raw ovine milk produced on three farms (4.16%) failed to meet the regulatory limit of 6.18 log_10_ CFU/mL, probably due to failures in implementing good hygiene and production practices. Comparable data on the recorded TMB counts have been reported in previous studies. In neighboring Hungary, the levels of bacteria in bulk tank milk on two out of three investigated farms exceeded the regulatory limit (7.4 and 6.3 log_10_ CFU/mL, respectively) [5]. Furthermore, in Switzerland, Spain, Egypt, and Greece, the bacteriological quality monitoring of ovine milk from different farms indicated mean TMB values of 6.05 log_10_ CFU/mL [24], 5.49 log_10_ CFU/mL [3], 6.30 log_10_ CFU/mL [25], and 5.48 log_10_ CFU/mL [26], respectively, without any reference to the level of contamination of individual samples. Similar to our findings, an investigation conducted by Muehlherr et al. [27] in Switzerland found that the recorded median TMB value for ovine milk was 4.78 log CFU/mL. In Romania, a study conducted by Caraba and Caraba [28] investigating the effects of the feeding management system on the milk production quality from sheep of the Turcana breed found that the TMB values recorded in different farms varied between 2.1 and 3.2 log_10_ CFU/mL. However, the resulting differences between the registered bacterial load of raw ovine milk in studies conducted in different countries can be markedly influenced by several factors, including milking practices, the farm [29], and the farm location [4]. Furthermore, different studies reported the microbiological quality of raw ovine milk exceeding regulatory limits, underlining the importance of implementing good hygiene and production practices, especially at the primary production level of the food chain. Nevertheless, the recorded data on the TMB count cannot provide detailed information about the amount of lactic acid, spoilage, or pathogenic bacteria, considering that contamination occurs in the same way for both pathogenic and non-pathogenic strains [6]. Likewise, even if the samples with a lower TMB count are expected to be safer, the occurrence of foodborne pathogens in this kind of milk has been previously demonstrated in several studies [30,31,32,33]. The significantly higher value recorded for the TMB count in May compared with April in 2024 can be associated with the gradual increase in the average air temperature with the approach of the summer season, which can greatly contribute to microbial multiplication in milk.

In this survey, *E. coli* was found in 66.6% (48/72) of the raw ewe milk samples tested, with an average contamination level of 2.47 log_10_ CFU/mL. The occurrence of the *stx*1 and *stx*2 virulence genes among the recovered isolates was 0.0% and 18.8%, respectively. The monitoring of the *E. coli* count as the main indicator of unfavorable hygienic conditions and fecal contamination of the raw ovine milk, associated with the evidence of infectious potential related to the toxin production of the isolated strains, has constituted the objectives of several investigations, but their number is still limited in the scientific literature. A previous study in Romania detected a higher rate of 75% (12/16) for *E. coli* in ovine milk-origin cheese assortments, traditionally produced and marketed by farmers directly to the consumer [2], alongside the presence of a single strain harboring the *stx*2 gene. Conversely, Momtaz et al. [13] published a lower contamination rate with *E. coli* (14.3%; 14/98) in raw ovine milk samples harvested from the bulk tanks of milk collection centers in Iran, alongside the occurrence of three isolates and one isolate containing the *stx*1 and *stx*2 genes, respectively. In other studies, *E. coli* was detected in 11.4% (4/35) of the examined raw ovine milk samples in Egypt [25]; in 61% (227/372) and 17.4% (131/752) of bulk tank milk specimens from the Lazio region of Italy [6] and the Castile-León region of Spain [34], respectively; and 40% (10/25) and 48% (12/25) of the tested conventional and organic farm milk samples from the Thessaly region of Greece, respectively [35]. In the same country, the examination of 595 bulk milk samples resulted in the isolation of *E. coli* serogroup O157 in 0.8% (*n* = 5) of the tested specimens, and 0.5% (*n* = 3) of them were Shiga toxin-positive isolates [36]. Furthermore, a high prevalence rate of 71.4% (45/63) was determined for the *Enterobacteriaceae* counts in bulk tank ovine milk samples tested in Switzerland [27], without any direct reference to the frequency of isolation of *E. coli* strains within the contaminated samples. However, in the same study, 12.7% of the molecularly examined Shiga toxin-producing *E. coli* strains harbored the *stx*2 gene [27]. Altogether, the presence of the stx2 gene in the milk-origin *E. coli* strains is unsurprising, considering that ruminants, especially cattle and sheep, are considered the main reservoirs of Shiga toxin-producing *E. coli* strains characterized by the production of potent cytotoxins, excreting these bacteria via feces and contaminating the external environment, including raw milk [13,25,27]. Nevertheless, considering the extremely variable results presented in the aforementioned studies, which can be markedly influenced by several factors (e.g., the study design, milking practices, flock size, housing conditions), it is difficult to integrate the positive findings within a harmonized preventive risk-based program.

In this investigation, the results of antimicrobial susceptibility monitoring of *E. coli* strains revealed considerable resistance against some drugs (AMP, TIM, LEX, and GEN) and a decreased (AMC and SXT) or total lack of (IMP) resistance to others. Limited data about the AMR profile of ovine milk-origin *E. coli* strains are available in the scientific literature for comparison. The studies conducted in different continents and countries used variable approaches to monitor the AMR of the isolated strains. A study conducted in Greece by Malissiova et al. [35] showed that 18 out of 22 milk-origin *E. coli* strains (82%) isolated from different conventional sheep farms were pansusceptible to a panel of 20 antimicrobials, and another 4 (18.2%) expressed resistance only against AMP. Moreover, the findings recorded in another study carried out in Jordan highlighted considerable resistance percentages toward folate pathway inhibitors (SXT; 57.8%) and aminoglycosides (GEN; 31.1%) [12]. In addition, in the same country, more than 30% of the isolated *E. coli* strains were resistant to AMP, and up to 20% of the isolates exhibited resistance toward GEN and SXT [37]. Likewise, the authors of an Iranian study indicated that, out of 102 ovine milk-origin *E. coli* strains, all expressed resistance to one or more antimicrobial agents, and the resistance percentages against SXT, AMP, and GEN were 31.37%, 26.47%, and 19.6%, respectively [13]. The recorded results and their comparison between different studies largely depend on the implementation of antimicrobial policy in animal husbandry, referring to all of the necessary strategies undertaken to organize the antimicrobial treatment to obtain animal health outcomes.

Notably, 5 (10.4%) out of the total of 48 tested isolates expressed MDR. These findings may reflect the undesired outcome of the repetitive, intensive, or irrational use of antimicrobials by veterinary practitioners to prevent and/or control infections in ovine populations in the screened region of Romania. However, this MDR rate is considerably lower than that reported for the cow milk-origin *E. coli* (23.3%) [2] or *S. aureus* (49%) strains in the same area [38]. Contrary to the obtained results in our study, none of the sheep milk-origin *E. coli* strains were characterized as MDR in Greece [39]. Nevertheless, 41.3% of the isolated *E. coli* strains from milk originating from sheep treated for mastitis were recorded as MDR in the same country [40]. Moreover, the recorded intermediate resistance level toward AMC in the case of 64.6% of the tested strains suggests an uncertain therapeutic effect concerning the administration of this drug. This finding also recommends the administration of a higher therapeutic dose than the recommended one, contributing to the appearance of the AMR phenomenon.

## 5. Conclusions

This study’s results indicate variable and appropriate TMB values in 95.8% of the tested bulk tank milk samples collected from the 72 ovine farms involved in this study, with 3 (4.2%) farms exceeding the regulatory limit of 6.18 log10 CFU/mL. Moreover, 66.6% of the samples contained *E. coli*, and 18.8% were highlighted as having pathogenic potential. These results indicate hygienic sanitary deficiencies, especially during the milking process, and constitute a potential public health hazard. The tested *E. coli* strains expressed AMR against most of the tested antimicrobials, with MDR recorded in a few cases, suggesting the need for implementation of an efficient antimicrobial stewardship program based on the urgent limitation of antimicrobial usage in sheep farms. Further investigations processing a higher number of samples and focusing on molecular evidence of other virulence factors/genes alongside the characterization of the genotypic AMR pattern of the *E. coli* isolates, which were limitations of this survey, are necessary to better understand the risk associated with ovine milk consumption.

## Figures and Tables

**Table 1:** Aerobic mesophilic bacteria count (TMB) and contamination levels with *E. coli*, carrying virulence genes, of the examined raw ovine milk. Bacteria count values are expressed in log_10_ CFU/mL.

Year	Month (No. Examined)	TMB Count			*E. coli*					
				Average (±Standard Deviation)	No. of Positive Samples (%)	Count			No. of Isolates Harboring Virulence Genes (%)	
		Min	Max			Min	Max	Average (±Standard Deviation)		
									*stx1*	*stx2*
2023	April (*n* = 11)	4.52	6.53	5.50 (±0.55)	7 (63.6)	2.36	2.82	2.60 (±0.19)	0	1
	May (*n* = 21)	4.25	6.87	5.29 (±0.64)	17 (81)	1.94	3.61	2.62 (±0.43)	0	3
2024	April (*n* = 19)	3.32	7.22	4.17 (±0.98)	4 (21.1)	1.82	2.38	2.02 (±0.37)	0	2
	May (*n* = 21)	4.12	6.05	5.18 (±0.45)	20 (95.2)	1.0	3.42	2.38 (±0.74)	0	3
Overall				4.99 (±0.86)	48 (66.6)			2.47 (±0.56)	0	9 (18.8)

**Table 2:** Categorization of different *E. coli* isolates recovered from raw ovine milk according to their AMR profiles.

No. of Isolates	AMR		Classification
	Pattern	Classes	
5	AMP + TIM + LEX + GEN	β-lactams, cephalosporins, and aminoglycosides	Multidrug-resistant
4	AMP + AMC + LEX	β-lactams and cephalosporins	Drug-resistant
1	AMP + TIM + SXT	β-lactams and sulfonamides	Drug-resistant
3	AMP + TIM	β-lactams	Drug-resistant
9	AMP	β-lactams	Drug-resistant
5	GEN	Aminoglycosides	Drug-resistant

Legend: AMP—ampicillin; TIM—ticarcillin–clavulanic acid; LEX—cefalexin; GEN—gentamicin; AMC—amoxicillin–clavulanic acid.

## Data Availability

All data generated or analyzed during this study are included in the submitted version of this manuscript.
